# Kinematics of Rotation in Joints of the Lower Limbs and Pelvis during Gait: Early Results—SB ACLR Approach versus DB ACLR Approach

**DOI:** 10.1155/2015/707168

**Published:** 2015-04-01

**Authors:** Andrzej Czamara, Iga Markowska, Aleksandra Królikowska, Andrzej Szopa, Małgorzata Domagalska Szopa

**Affiliations:** ^1^The College of Physiotherapy in Wrocław, ul. Kościuszki 4, 50-038 Wrocław, Poland; ^2^The Center of Rehabilitation and Medical Education, ul. Kościuszki 4, 50-038 Wrocław, Poland; ^3^School of Health Sciences, Medical University of Silesia, Medyków 12, 40-752 Katowice, Poland

## Abstract

It is difficult to find publications comparing rotation kinematics in large joints of the lower limbs and pelvis during gait in patients after single-bundle (SB) reconstruction of the anterior cruciate ligament (ACLR) with double-bundle (DB) ACLR of the knee. The aim of this study was to compare rotation kinematics in ankle, knee, and hip joints and the pelvis during gait in the 14th week after SB and DB ACLR. The subjects were males after SB (*n* = 10) and DB (*n* = 13) ACLR and a control group (*n* = 15). The values of kinematic parameters were recorded during internal (IR) and external (ER) rotation in the joints during gait using the BTS SMART. The SB ACLR group obtained significantly higher values of ER in the involved knee comparing to DB ACLR and controls and excessive IR in the hip comparing to controls. In the DB ACLR group, excessive ER was noted in the involved leg's foot. Comparing with the DB ACLR and control groups, SB ACLR subjects had more substantial disorders of rotation kinematics in the lower limb joints. However, in both ACLR groups, 14 weeks of postoperative physiotherapy were not enough to fully restore rotation kinematics in joints of the lower limbs during gait.

## 1. Introduction

The goal of double-bundle reconstruction of the anterior cruciate ligament (DB ACLR) was to obtain a rotational stability of the tibia better than that achieved using a single-bundle reconstruction (SB ACLR) [1, 2]. Similar opinions have been presented by Hantes et al. and Hemmerich et al. [3, 4]; by contrast, however, Meredick et al. and Song et al. did not confirm this hypothesis [5, 6]. Although Claes et al. reported recovery of correct kinematics of tibial rotation six months after SB ACLR and DB ACLR, they did not show the DB ACLR approach to be any better than SB ACLR regarding effects on gait kinematics and other dynamic physical activities [7]. Tsarouhas et al. did not report any differences in tibial rotation and peak torque between SB ACLR and DB ACLR groups during gait or while walking up and down stairs; however, peak torque values obtained from the involved legs were lower in both groups compared with the values obtained from the uninvolved side [8]. Scanlan et al. found higher values reflecting tibial rotation in the stance phase of gait in the involved legs in comparison with those obtained from the uninvolved legs 2 years after ACLR [9]. To date, there has been no comparative analysis of tibial rotation kinematics during gait with simultaneous changes in the rotation of the feet, tibia, femur, and pelvis in SB ACLR and DB ACLR groups.

The goal of this study was the assessment and comparative analysis of tibial rotation associated with changes in large joints of the lower limbs and pelvis during gait in patients after DB ACLR and SB ACLR procedures.

## 2. Materials and Methods

All subjects were informed about the goal of the study and the approach to be used. The study was approved by the local ethics committee, and written informed consent forms were signed by all of the participants prior to the study. The study was conducted according to the ethical guidelines and principles of the Declaration of Helsinki.

### 2.1. Materials

**Figure 1:**
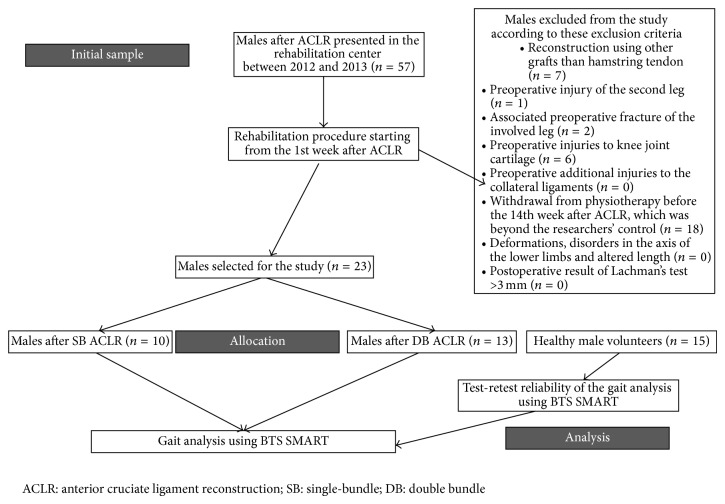
Flowchart of the study.

The sample comprised 23 males who had undergone ACLR using an ipsilateral graft of hamstring muscles. The subjects were recruited to each group based on the type of ACLR (Figure 1).

The SB ACLR group (*n* = 10) comprised males who underwent SB ACLR, and the DB ACLR group (*n* = 13) consisted of males with a history of DB ACLR. In addition, 15 participants without known cardiovascular or orthopedic problems (control group) were recruited from the student population of the college where the study was conducted. Up to 4 weeks postoperatively patients from the SB and DB ACLR groups were taking anticoagulants and analgesics. Sport-related activity in all participants before the injury was scored as 6-7 points on the Tegner Activity Scale. The average time period between complete anterior cruciate ligament tear and the surgery was 4 (±2) months. Characteristics of the studied sample are presented in Table 1.

### 2.2. Surgical Procedures

#### 2.2.1. Single-Bundle ACL Reconstruction

The semitendinosus (ST) and gracilis (GR) tendons were harvested through a 25–30 mm oblique incision over pes anserinus, which had been harvested using a tendon striper. The tendons were prepared as a 4-stranded double-looped hamstring autograft. The ACL tibial guide (Aesculap, Smith&Nephew, Artherx) was set to 45° (from the medial tibial cortex to the center of footprints) to prepare the tibial tunnel. Tunnel diameter was determined according to the harvested tendon diameter, which varied from 7 to 8 mm [10]. The femoral tunnel made through an anteromedial portal with knee flexion at a minimum 120° was placed on the posterior aspect of the notch, at approximately 10:30 o'clock orientation for the right knee or at approximately 1:30 o'clock orientation for the left knee [10]. Each graft was introduced through the tibial tunnel to the femoral tunnel and fixed on the lateral femoral cortex by flipping the EndoButon. The graft was manually tensioned at 30° of knee flexion, and the tibial side was fixed with a bioabsorbable screw (BIORCI; Smith&Nephew Endoscopy).

#### 2.2.2. Double-Bundle ACL Reconstruction

**Figure 2:**
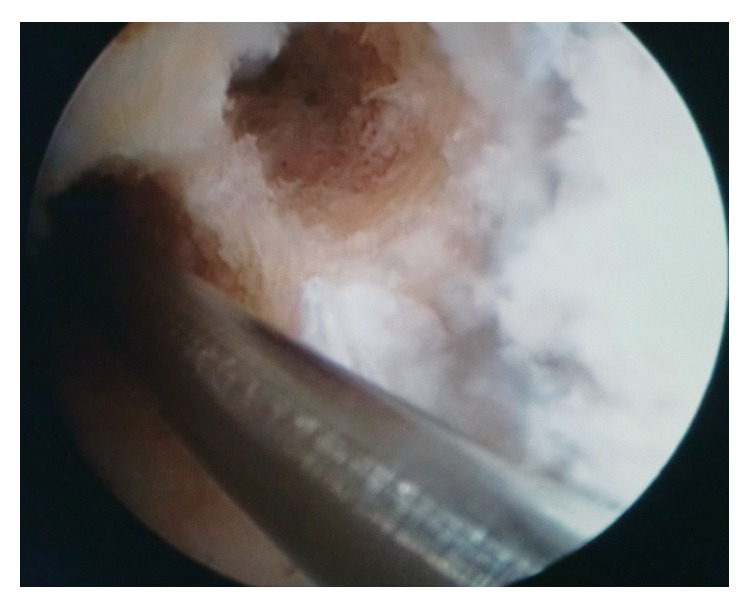
Double-bundle ACL reconstruction. Femoral posterolateral bundle (K-wire in) and anteromedial bundle tunnels.

**Figure 3:**
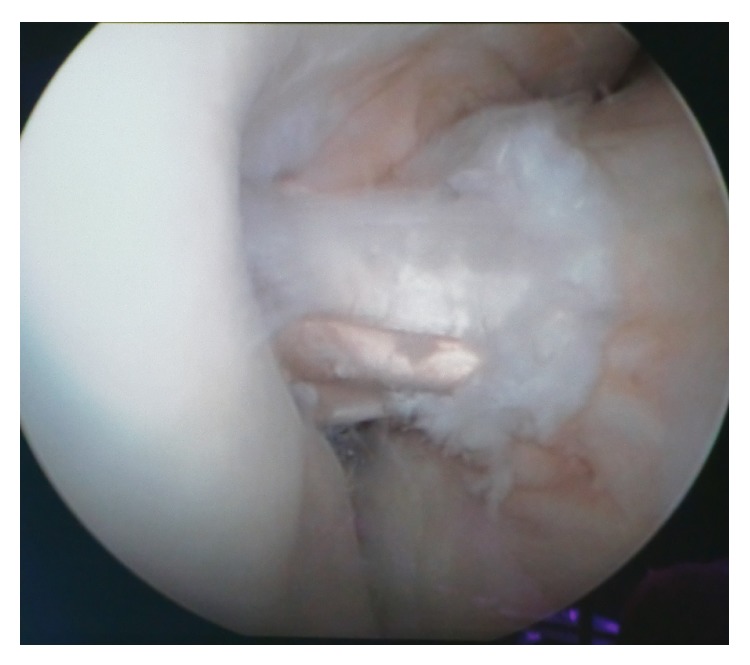
Double-bundle ACL reconstruction. Anteromedial and posterolateral grafts.

**Figure 4:**
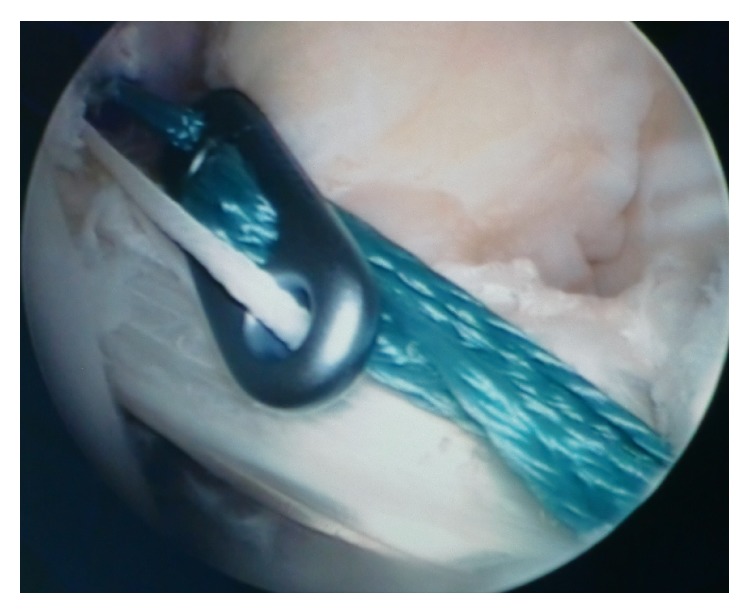
Double-bundle ACL reconstruction. The introduction of anteromedial bundle.

**Figure 5:**
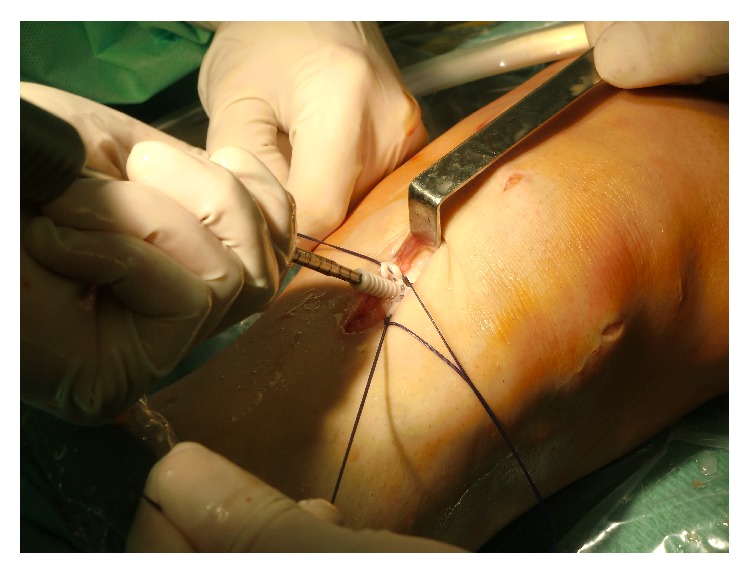
Double-bundle ACL reconstruction. Tibial fixation of the posterolateral bundle.

**Figure 6:**
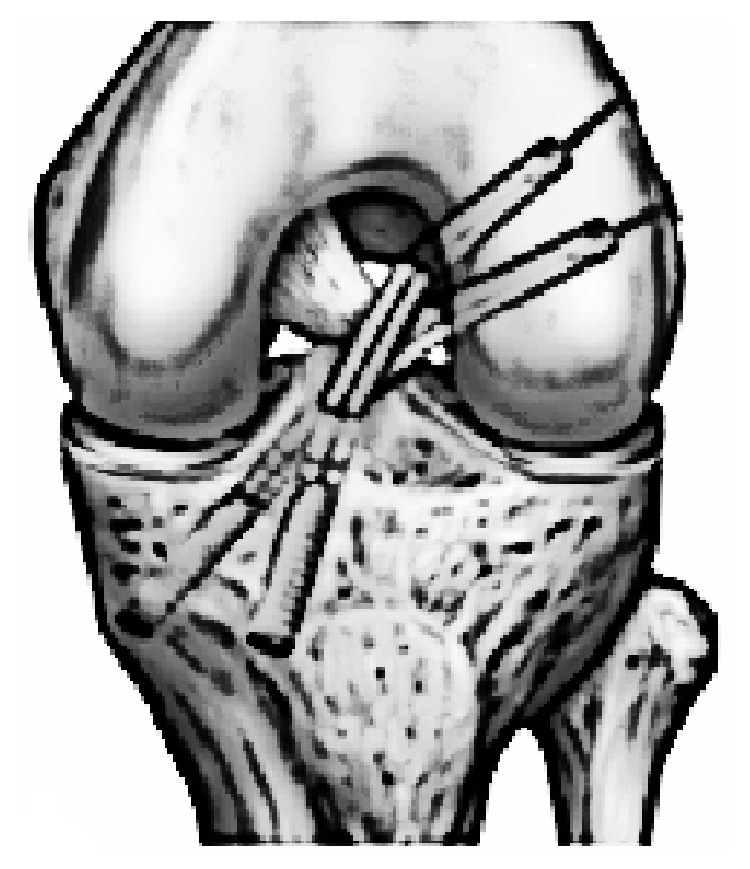
Scheme of the double-bundle ACL reconstruction.

The ST and GR tendons were harvested through a 25–30 mm oblique incision over pes anserinus, which had been harvested using a tendon striper. The tendons were prepared as a two hamstring autografts. The anteromedial (AM) graft was generally made slightly larger than the posterolateral (PL) graft. The AM bundle was created with the use of the doubled semitendinosus graft, resulting in 6-7 mm graft diameter. The PL bundle was created using the doubled gracilis graft, resulting in 5-6 mm. Two guide pins were inserted from the medial tibial cortex to the center of the AM and PL bundle footprints with a drill guide system (Smith&Nephew, Artherx). Firstly, the tibial tunnel for the AM bundle was drilled. An ACL tibial drill guide was placed on the AM aspect of the ACL footprint with the angle set to 55°. The starting point of the AM tibial tunnel was the same as in standard SB ACL technique. The PL tibial guidewire was placed on the PL aspect of the ACL tibial footprint with the angle set to 55°. An osseous bridge of the 1 to 2 cm was preserved on the tibial cortex between the two bone tunnels. Tunnel diameter was determined according to the harvested tendon diameter, which varied from 6 to 7 mm AM and that of the PL tunnel 5-6 mm [11, 12]. With use of anterolateral entry femoral aimer (S&N), the 2.4 mm guide pins were separately inserted from the outside into each portion of the PL and AM bundle footprints behind the “ridge" and anterior to the cartilage margin. For PL bundle femoral tunnel creation was used to mark the femoral target point. The target point was located at the center of the PL bundle footprint or approximately 6 mm anterior (high) to the posterior margin of the osteochondral junction at 90° flexion to mark the drilling spot [11]. The knee was then flexed 110° to 130° and the K-wire drilling femoral tunnel. After the K-wire was overdrilled through the femur using 4.5 mm EndoButton drill (S&N), tunnel length was measured and the femoral tunnel was created using a cannulated drill with a diameter the same size as the graft diameter. The AM bundle tunnel was created using the same technique employed in PL bundle tunnel creation. The femoral target point was at 1:30 (or 10:30) o'clock with respect to the notch apex and at the midpoint between the resident's ridge and posterior cortex. Once the insertion point was marked at 90° flexion, the knee was flexed at 110 to 130° and the tunnel was created. The bony wall between both femoral bone tunnels should be a minimum of 2 mm (Figure 2). Both grafts were inserted from distal to proximal via the tibial tunnels into the femoral tunnels (Figure 3). The PL graft passed first followed by the AM graft (Figure 4). The 5.0 to 7.0 mm tunnels were created for the AM and PL grafts. Each graft was introduced through the tibial tunnel to the femoral tunnel (separately, firstly PL bundle and secondly AM bundle) and fixed on the lateral femoral cortex by flipping the EndoButon. The PL graft was manually tensioned at 20° of knee flexion, AM graft was manually tensioned at 30° of the knee flexion, and the tibial side was fixed with a bioabsorbable screw (BIORCI; Smith&Nephew Endoscopy) (Figures 5 and 6).

### 2.3. Orthopedic Examination

The orthopedic examination comprised assessment of pain, based on the visual analog scale and the patient's subjective assessment of stability. The medical examination consisted of clinical tests: Lachman's test and tests to assess stability in posterior cruciate ligaments, collateral ligaments, menisci, the patellofemoral joint, Q-angle, and axis of the lower limbs. Moreover, the circumferences and mobility of joints were compared [13].

### 2.4. Postoperative Physiotherapy

Between the first week after ACLR and the tests conducted in SB ACLR and DB ACLR groups, the same physiotherapeutic procedures were carried out by one physiotherapist at the same rehabilitation center. All patients were instructed on how to perform additional exercises at home. Postoperative procedures were based on the physiotherapy protocol for patients after ACLR [14].

### 2.5. Recording and Measurements of Rotation Kinematics Parameters

**Figure 7:**
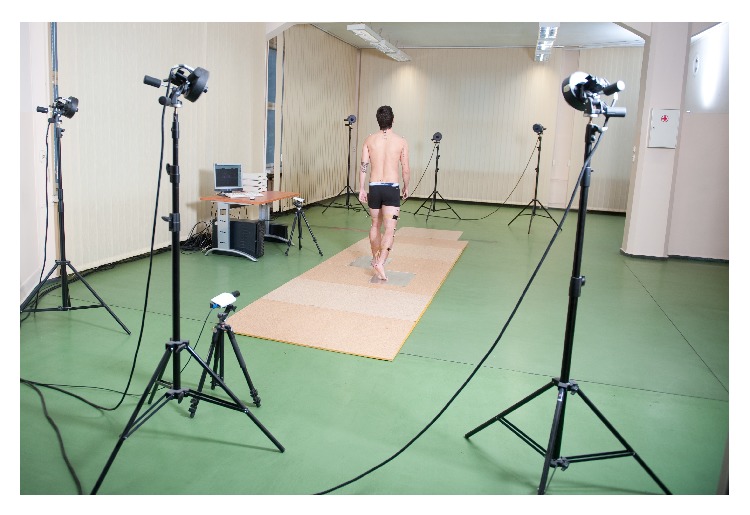
Recording and measurements of rotation kinematics parameters during gait using BTS Smart system.

The parameters of rotation kinematics were measured during gait in individual joints of the lower limbs and the pelvis using the optoelectronic BTS SMART system (BTS Bioengineering, Milan, Italy) for three-dimensional record and analysis of movement. Six infrared camcorders with a sampling frequency of 120 Hz were used for recording. Two Kistler platforms were used for the measurement of ground reaction forces for individual components (sampling frequency 960 Hz). The methodology of measurements was in accordance with the manufacturer's instructions [15]. Anthropometric measurements of the right side and, next, the left side of the body were performed. The body height and body mass were measured in the studied groups, in addition to the width of the knee joints and the ankle joints and the absolute length of the lower limbs. After cleansing of the skin, 22 markers were placed on the left and the right leg at the levels of the calcaneal tuberosity, head of the fifth metatarsal bone, lateral malleolus, head of fibula, half length of the shin, lateral condyle of the femur, half length of the femur, area of the greater trochanter, anterior superior iliac spine, spinous process of 1st sacral vertebra and 7th cervical vertebra, and acromia (left and right upper limb) [16] (Figure 7). The participant walked six times unsupported at his normal speed, covering the distance of 7-8 m, starting each walk with the left leg. The data collected during the examination were then processed using BTS SMART (BTS Capture, Tracker, Analyzer) [15, 16]. From all of the recorded walks, the two best were selected (hitting the piezoelectric platform and readout of all the markers on the patient's body by the system during gait). The range of rotation movement in the ankle, knee, and hip joints and the pelvis was analyzed during six phases of gait cycle: the first contact of the foot with the ground (KHS), followed by two events concerning the knee joint, involving recording of the parameters obtained from the remaining joints, maximal flexion of the knee at the beginning of the stance phase (K1), maximal extension of the knee during a single stance (K2), the moment when the toes were detached from the ground at the end of stance phase (KTO), the moment of maximal knee joint flexion during the swing phase (K3), and maximal extension of the knee joint during the preparatory phase preceding the stance phase (K4).

Measurements of the kinematic parameters of gait in the SB ACLR and DB ACLR groups were performed at the end of the 14th week following ACLR. In the control group, the test was repeated twice for test-retest reliability.

### 2.6. Test-Retest Reliability

The test-retest reliability of the gait analysis using the BTS SMART system was evaluated to validate the use of the system in clinical research. The testing was conducted in two identical sessions held every three days at the same time. All measurements were performed by the same researcher. During both sessions, gait analysis was performed in the same way. In the control group, the test was performed using the same methodology as that reported in Section 2, describing the recording and measurements of the kinematic rotation parameters during gait. The results of an intraclass correlation coefficient (ICC) test for ankle joint rotation movement for the six studied gait phases, between the first and the second test, ranged from 0.87 to 0.98, which indicates high and very high coefficient of study repeatability. Similarly, the values obtained from the ICC test were very high for most of the gait phases and ranged from 0.82 to 0.99 for the knee joint. Moreover, a value reflecting very high repeatability was obtained for the hip joint, with ICC results ranging from 0.89 to 0.96, and for the pelvis, with the corresponding values ranging from 0.62 to 0.91. For most of the samples, ICC values obtained for the pelvis exceeded 0.82.

### 2.7. Statistical Analysis

The mean value (*x*) and standard deviation (SD) were calculated for the obtained test results. To analyze the distribution, the Shapiro-Wilk test was applied [17, 18]. For the intragroup comparison of results obtained by lower limbs the parametric *t*-test for dependent samples was applied. The involved legs (SB ACLR group and DB ACLR group) were compared with the values obtained in right legs in the control group in the measurement during the first session. The uninvolved legs in groups after ACLR were compared to left ones (control group). For comparison of the values obtained from the three studied groups (SB ACLR, DB ACLR, and control), analysis of variance (ANOVA) was applied. Moreover, a post hoc comparison using Tukey's test was conducted. Statistical significance was set at *P* < 0.05. ICCs (Shrout and Fleiss model 2) were calculated to compare the data between sessions in the test-retest assessment [19]. The following guidelines, described by Cicchetti and Sparrow, were used to assess reliability coefficients: less than 0.40 was considered poor, 0.40–0.59 was considered fair, 0.60–0.74 was considered good, and 0.75 or greater was considered excellent [20].

## 3. Results

### 3.1. Ankle Joint

Comparative analysis of rotation kinematics in ankle joints of the involved legs, including comparison of the values obtained from SB ACLR, DB ACLR, and control groups, did not show statistically significant differences in all six studied phases of gait during the 14th week postoperatively (Table 2). By contrast, the intragroup analysis showed excessive external rotation of the foot in the involved leg in the DB ACLR group, particularly in KHS, K1, and K3 phases, compared with the uninvolved side (Table 2).

### 3.2. Knee Joint

The intragroup analysis showed no differences between the results of involved legs and uninvolved legs in the SB ACLR and DB ACLR groups (Table 3). The comparison of the results of involved legs in SB ACLR and DB ACLR groups and right leg in control group showed statistically significant differences in the 5 gait cycle phases: KHS, K1, KTO, K3, and K4 (Table 3). Statistically significant differences were also found in the comparison of the results of uninvolved legs in SB ACLR and DB ACLR groups and left leg in control group during phases K1 and KTO (Table 3). The post hoc Tukey test showed persistent excessive external rotation in the involved knee joint during KHS, K1, KTO, K3, and K4 phases in the SB ACLR group in comparison with the control group; the corresponding value was statistically significant (Table 4). Moreover, in the SB ACLR group the significant excessive external rotation was maintained in the involved knee, in comparison with the results obtained from the involved knee joint in the DB ACLR group for KTO and K3 phases (Table 4). The post hoc test also revealed statistically significant differences between results of uninvolved leg in SB ACLR group and left leg in control group for phases K1 and KTO (Table 4).

### 3.3. Hip Joint

There were no statistically significant differences between the involved and uninvolved side in the SB ACLR and DB ACLR groups (Table 5). However, the results of ANOVA test comparing obtained results in involved legs in groups after ACLR and the right leg in control group showed statistically significant differences in hip rotation during all of the gait cycles (Table 5). The comparison of the results obtained in uninvolved legs and left leg showed statistically significant differences in phases K1, KTO, K3, and K4 (Table 5). The post hoc Tukey test revealed statistically significant excessive internal rotation in the hip joint at the side of the involved leg for all studied cycles of gait (from KHS to K4 phase) in the SB ACLR group in comparison with the control group (Table 6). In the DB ACLR group, no statistically significant changes were noted in the studied joints in comparison with the results obtained from the control group (Table 6). The post hoc Tukey test also showed statistically significant excessive internal rotation in the hip joint at the side of the involved leg for phases K1, KTO, K3, and K4 in the SB ACLR group in comparison with the control group (Table 6). In the DB ACLR group, statistically significant changes were noted in the studied joints in comparison with the results obtained from the control group in phase KTO (Table 6).

### 3.4. Pelvis

Also comparative analysis of rotation kinematics in the pelvis between SB ACLR and DB ACLR groups did not show any statistically significant differences for studied phases of gait (Table 7). There were no statistically significant differences between the involved and uninvolved side (Table 7). No statistically significant differences were found between groups after ACLR and the control group in the values corresponding to pelvis rotation (Table 7).

## 4. Discussion

A simultaneous comparative analysis of rotation kinematics in the ankle, knee, and hip joints and the pelvis was conducted for the six studied phases of gait cycle in SB ACLR and DB ACLR patients. It was found that 14 weeks of supervised physiotherapy for patients after ACLR did not restore the baseline rotation kinematics in the ankle, knee, and hip joints while walking on a flat surface. The results indicate that, compared with the SB ACLR and control groups, the DB ACLR group presented with fewer pronounced disorders of rotation kinematics in the studied joints of the lower limbs during gait. The study also suggests that analyzing tibial rotation in the knee joint with simultaneous changes in rotation in large joints of the lower limbs provides better opportunities than singular analysis of rotation in the knee joint for the assessment of disorders in gait kinematics.

In practice, the significance of such studies is unquestionable during the early period following SB and DB ACLR. Gao and Zheng (2010) showed that the kinematic parameters of gait are not fully restored in patients after ACLR compared with control values [21]. Interesting comments concerning the assessment of the early and remote analysis of gait kinematics in the knee joints are presented by Webster et al. (2011, 2012) who have found that with time, kinematic parameters of rotation in the knee joint improve in patients after ACLR between 6 months and two years after the surgery [22, 23]. The authors showed that long-term test results provide considerably more clinical information on changes occurring during locomotion in patients compared with early results obtained after ACLR [22, 23]. On the other hand, early studies on rotation kinematics are justified, given the surgical procedures applied thus far in addition to the current postoperative protocols of physiotherapy. One may hypothesize that implementation of closely defined postoperative procedures after ACLR may affect the remote results of gait kinematics. Confirmation of this hypothesis will require both early and remote studies and analyses. The data presented in the literature indicate that it is possible to restore kinematic parameters of the knee joints in the sagittal plane while walking on a flat surface as early as 10–12 weeks following ACLR [24]. On the other hand, in a systematic review, Gokeler et al. (2013) suggested that altered gait patterns in patients after ACLR can persist for up to five years after surgery [25]. The authors emphasized that rehabilitation techniques should be examined in order to minimize changes in knee biomechanics during gait, as they have the potential to impact the development of osteoarthritis [25].

Some studies have shown that, between the 13th and 16th week after ACLR, the peak torques in muscles responsible for stability and dynamic functions of the knee joints are not completely restored in the sagittal or the transverse plane [14, 26]. The results of these studies suggest that it should be ascertained whether the maintained limitation of the level of peak torque during the 14th week after ACLR can affect kinematic parameters of gait. The between-subject analysis performed in the present study showed excessive external rotation in the involved knee joint in the SB ACLR group in comparison with the DB ACLR group. Also kinematic tests performed two years after ACLR by Lee et al. (2012) suggested that DB ACLR improved knee rotatory laxity at 30° and 60° of flexion better than SB ACLR [27]. In the present study in DB ACLR group, excessive foot rotation was noted at the involved side in three of the six studied phases of gait cycle in comparison with the uninvolved side. Comparative analysis of results between SB ACLR and DB ACLR groups showed differences between the knee joint and the hip joint. An excessive external rotation in the involved knee joints and excessive internal rotation in the hip joints at the involved side for most of the studied phases of gait cycle in the SB ACLR group when compared with the control group were noted. Moreover, in the SB ACLR group, persistent excessive rotation was noted in the involved knee joints for KTO and K3 phases, compared with the results obtained from DB ACLR subjects. The results presented herein can be complementary to the comparative assessment of SB ACLR and DB ACLR techniques, most often analyzed from the clinical point of view embracing surgical techniques, types of grafts, graft placement techniques, and graft fixation techniques [1, 2, 11, 28–32]. The results of present study additionally provide detailed information on the disorders of rotation kinematics in the studied joints during locomotion. The values obtained indicate that, in the DB ACLR group, the physiotherapy program should also include exercises aimed at improvement of muscle function in the involved leg of muscles responsible for internal rotation of the foot during gait and when the foot makes contact with the ground (KHS), during maximal flexion of the knee joint in the stance phase (K1), and during maximal flexion of the knee joint in the swing phase (K3) to reduce excessive external rotation of the foot at the involved side. For the SB ACLR group it is necessary to introduce specific exercises aimed at improvement in the function of muscles responsible for internal rotation in the involved knee, because of persistent excessive external tibial rotation in the involved knee joint in comparison with the uninvolved side and the values obtained from the control group. In the SB ACLR group it is also necessary to introduce exercises aimed at improving the function of muscles responsible for external rotation in the hip joint.

Persistent disorders of rotation kinematics in the studied parts of lower limb joints during gait in both groups of patients suggest the necessity of continuing postoperative physiotherapy, especially performance of additional exercises aimed at improving coordination and stability when the foot makes contact with the ground. Exercises stimulating improvement of the function of muscles affecting the foot, the tibia, and the femur, as well as the pelvic girdle and the trunk, should be continued. This approach is in accordance with the principles of introducing specialist postoperative physiotherapy after ACLR. Moreover, elements of functional training with graded difficulty of motor tasks should be introduced in patients while performing different kinds of locomotor activities, neuromuscular coordination, and dynamic proprioception. During these stages, resistance eccentric-concentric training and many different elements of kinesiotherapy are introduced to restore optimal physical fitness levels [14]. Some authors recommend using high-intensity resistance training during rehabilitation after ACLR as it can improve muscle power without adverse effects on joint laxity [33]. There are also articles showing benefits of using neuromuscular electrical stimulation as an element of rehabilitation after ACLR [34].

Monitoring of the responses from the knee joints is presently insufficient, owing to the fact that analysis of rotations occurring only in knee joints does not reflect all of the multiarticular disorders of gait kinematics. To date, there have been no reports on this topic in the literature. The available articles by Claes et al., Tsarouhas et al., Scanlan et al., and Georgoulis et al. [7–9, 35] report rotation in knee joints after ACLR. Claes et al. (2011) found that 6 months after ACLR SB and DB techniques adequately restore tibial rotational excursion while plain walking and while low- and high-demand motor tasks including pivoting [7]. Tsarouhas et al. (2011) noted that high-intensity activities combining stair ascending or descending with pivoting produce similar tibial rotation in SB and DB ACL-reconstructed patients. During such manoeuvres, the reconstructed knee may be subjected to significantly lower rotational loads compared with the intact knee [8]. Scanlan et al. (2010) found abnormal rotation of the tibia in the stance phase of gait in the involved legs 2 years after ACLR [9]. Georgoulis. et al. (2010) demonstrated that patients with anterior cruciate ligament-deficient knees experienced repeated episodes of rotational instability during walking, whereas patients with reconstruction experienced tibial rotation closer to normal [36].

The multiarticular biomechanical assessment of rotation kinematics in large joints of the lower limbs and the pelvis during gait is an objective tool to evaluate restored locomotion in ACLR patients. This study enabled us to assess not only the dynamic functions of the knee joints in the transverse plane but also dynamic rotations in other joints of the lower limbs and the pelvis during locomotion, and their reciprocal relationships. Moreover, in the future the results obtained from such studies can be referred, compared, and correlated with the results of clinical tests, such as tests of anterior tibial translation toward the thigh (Lachman's test and anterior drawer test), measurements of knee stability using arthrometers, and the pivot-shift test. The results obtained when the research conception is presented can also be used as an objective tool to verify patients' self-assessment of the level of restored locomotion, usually using standard questions included in the Lysholm knee-scoring scale, International Knee Documentation Committee (IKDC) Subjective Form, or other scales [1, 2, 32, 37–41]. Also, because, according to Kwon et al. (2015) in healthy individuals, the external rotation peak of the knee joint, as well as plantar flexion peaks of the ankle joint, increases with an increase of gait speed while walking down a walkway it would be interesting to correlate the results of the present study with gait speed in the three studied groups [42].

Our study has some limitations, such as the expensive apparatus used and the precise recording procedure, timing, and team training required to conduct the study. Moreover, a longer observation time is necessary for proper assessment of remote results of rotation in large joints of the lower limbs and the pelvis in cohorts undergoing ACLR using two different surgical techniques. What is more, functional scales as IKDC and clinical outcomes were not reported and it would be something else to do in a long term of the study.

## 5. Conclusions

- In both ACLR groups, the 14-week period of postoperative physiotherapy was too short to fully restore rotation kinematics in joints of the lower limbs during gait.
- In the SB ACLR group, more disorders of rotation kinematics were noted in large joints of the lower limbs during gait in comparison with the DB ACLR and control groups.
- In patients after ACLR, the analysis of tibial rotation in the knee joint with simultaneous changes in rotation in large joints of the lower limbs during gait provides better opportunities than singular analysis of rotation in the knee joint for the assessment of disorders in gait kinematics.

## Figures and Tables

**Table 1:** Participant characteristics and time since surgery.

Studied sample	Number of individuals	Age (years)		Body mass (kg)		Body height (cm)		Time elapsed from ACLR (weeks)		Involved leg		Dominant leg	
		*x*	SD	*x*	SD	*x*	SD	*x*	SD	R	L	R	L
SB ACLR	*n* = 10	26.00	10.22	82.20	9.19	178.70	8.39	13.60	3.27	*n* = 6	*n* = 4	*n* = 10	—
DB ACLR	*n* = 13	28.00	8.14	76.31	11.26	180.54	9.73	14.08	5.42	*n* = 9	*n* = 4	*n* = 11	*n* = 2
Control	*n* = 15	23.40	2.41	76.27	7.09	177.40	4.15	—		—		*n* = 12	*n* = 3
	*P*	0.233		0.231		0.550		—		—		—	

ACLR: anterior cruciate ligament reconstruction; SB ACLR: group after single-bundle ACLR; DB ACLR: group after double-bundle ACLR; control: control group; *x*: arithmetic mean; SD: standard deviation; R: right leg; L: left leg; *P*: significance level (one-way Anova).

**Table 2:** Intragroup evaluation and intergroup comparison of examined variables in the ankle joint.

Ankle joint						
Internal (+) and external (−) rotation during gait cycle (degrees)						
Gait phases	Studied group	Involved/right leg		Uninvolved/left leg		*P* ^∗^
		*x*	SD	*x*	SD	
KHS	SB ACLR	−9.94	5.62	−7.31	9.65	0.501
	DB ACLR	−12.45	5.89	−6.44	11.52	**0.046**
	Control	−13.30	3.57	−11.15	3.61	0.075
	*P *	0.262		0.323		

K1	SB ACLR	−10.78	5.19	−7.14	9.35	0.350
	DB ACLR	−13.05	6.63	−5.77	10.85	**0.015**
	Control	−13.01	4.23	−10.98	4.76	0.135
	*P *	0.534		0.256		

K2	SB ACLR	−13.22	4.84	−10.49	9.67	0.469
	DB ACLR	−15.26	6.08	−9.22	11.07	0.069
	Control	−17.22	4.16	−15.21	4.15	0.142
	*P *	0.165		0.164		

KTO	SB ACLR	−6.71	3.42	−2.03	7.55	0.097
	DB ACLR	−9.64	11.05	−3.38	12.15	0.311
	Control	−8.01	4.72	−5.88	3.50	0.084
	*P *	0.634		0.508		

K3	SB ACLR	−13.51	5.62	−8.00	10.65	0.165
	DB ACLR	−15.25	11.54	−7.08	13.91	**0.039**
	Control	−13.23	6.47	−14.51	7.59	0.387
	*P *	0.799		0.162		

K4	SB ACLR	−11.30	5.27	−9.62	10.45	0.673
	DB ACLR	−12.78	7.17	−7.20	12.84	0.263
	Control	−14.60	4.87	−13.35	4.35	0.398
	*P *	0.384		0.247		

ACLR: anterior cruciate ligament reconstruction; SB ACLR: group after single-bundle ACLR; DB ACLR: group after double-bundle ACLR; control: control group; *x*: arithmetic mean; SD: standard deviation; KHS: the first contact between the foot and the ground; K1: maximal knee flexion at the beginning of stance phase; K2: the biggest extension of the knee during a single stance; KTO: detaching the toes at the end of stance phase; K3: maximal knee flexion during swing phase; K4: maximal extension of the knee joint in the preparatory phase before stance phase; *P*: significance level (one-way Anova); *P* ^∗^: significance level (parametric *t*-test for dependent samples). Values in bold are statistically significant.

Presented values obtained in the SB and DB ACLR groups refer to involved leg while values obtained in the control group refer to the right leg.

**Table 3:** Intragroup evaluation and intergroup comparison of examined variables in the knee joint.

Knee joint						
Internal (+) and external (−) rotation during gait cycle (degrees)						
Gait phases	Studied group	Involved/right leg		Uninvolved/left leg		*P* ^∗^
		*x*	SD	*x*	SD	
KHS	SB ACLR	−21.85	8.80	−20.41	8.13	0.646
	DB ACLR	−15.38	8.52	−17.92	9.65	0.426
	Control	−13.79	3.38	−13.77	5.56	0.985
	*P*	**0.024**		0.114		

K1	SB ACLR	−15.57	9.92	−13.81	7.40	0.508
	DB ACLR	−9.12	8.46	−10.20	10.45	0.404
	Control	−4.83	3.46	−4.61	6.48	0.889
	*P*	**0.005**		**0.029**		

K2	SB ACLR	−16.71	11.16	−16.56	7.19	0.973
	DB ACLR	−11.94	8.35	−13.18	9.56	0.697
	Control	−10.24	3.50	−11.06	6.04	0.666
	*P*	0.135		0.230		

KTO	SB ACLR	−14.96	9.20	−13.64	8.28	0.732
	DB ACLR	−6.57	8.93	−8.29	8.73	0.618
	Control	−3.43	3.48	−2.31	5.67	0.455
	*P*	**0.002**		**0.003**		

K3	SB ACLR	−14.50	8.35	−11.95	11.30	0.564
	DB ACLR	−4.75	8.60	−6.92	11.78	0.550
	Control	−2.31	5.38	−4.73	10.10	0.272
	*P*	**0.001**		0.284		

K4	SB ACLR	−22.92	8.30	−19.88	7.42	0.484
	DB ACLR	−16.15	11.37	−17.58	9.82	0.701
	Control	−12.43	3.11	−12.65	6.62	0.892
	*P*	**0.012**		0.081		

ACLR: anterior cruciate ligament reconstruction; SB ACLR: group after single-bundle ACLR; DB ACLR: group after double-bundle ACLR; control: control group; *x*: arithmetic mean; SD: standard deviation; KHS: the first contact between the foot and the ground; K1: maximal knee flexion at the beginning of stance phase; K2: the biggest extension of the knee during a single stance; KTO: detaching the toes at the end of stance phase; K3: maximal knee flexion during swing phase; K4: maximal extension of the knee joint in the preparatory phase before stance phase; *P*: significance level (one-way Anova); *P* ^∗^: significance level (parametric *t*-test for dependent samples). Values in bold are statistically significant.

Presented values obtained in the SB and DB ACLR groups refer to involved leg while values obtained in the control group refer to the right leg.

**Table 4:** Intergroup comparison of examined variables in the knee joint (results of the post hoc test).

Tukey's test results (*P*)								
Gait phase	Studied group	Involved/right leg			Studied group	Uninvolved/left leg		
		SB ACLR	DB ACLR	Control		SB ACLR	DB ACLR	Control
KHS	SB ACLR	—	0.087	**0.022**	SB ACLR	—	0.731	0.109
	DB ACLR	0.087	—	0.822	DB ACLR	0.731	—	0.353
	Control	**0.022**	0.822	—	Control	0.109	0.353	—

K1	SB ACLR	—	0.110	**0.003**	SB ACLR	—	0.558	**0.026**
	DB ACLR	0.110	—	0.289	DB ACLR	0.558	—	0.190
	Control	**0.003**	0.289	—	Control	**0.026**	0.190	—

K2	SB ACLR	—	0.325	0.120	SB ACLR	—	0.554	0.201
	DB ACLR	0.325	—	0.835	DB ACLR	0.554	—	0.750
	Control	0.120	0.835	—	Control	0.201	0.750	—

KTO	SB ACLR	—	**0.027**	**0.001**	SB ACLR	—	0.223	**0.002**
	DB ACLR	**0.027**	—	0.503	DB ACLR	0.223	—	0.105
	Control	**0.001**	0.503	—	Control	**0.002**	0.105	—

K3	SB ACLR	—	**0.010**	**0.001**	SB ACLR	—	0.528	0.861
	DB ACLR	**0.010**	—	0.665	DB ACLR	0.528	—	0.257
	Control	**0.001**	0.665	—	Control	0.257	0.861	—

K4	SB ACLR	—	0.131	**0.009**	SB ACLR	—	0.778	0.085
	DB ACLR	0.131	—	0.456	DB ACLR	0.778	—	0.251
	Control	**0.009**	0.456	—	Control	0.085	0.251	—

ACLR: anterior cruciate ligament reconstruction; SB ACLR: group after single-bundle ACLR; DB ACLR: group after double-bundle ACLR; control: control group; *x*: arithmetic mean; SD: standard deviation; KHS: the first contact between the foot and the ground; K1: maximal knee flexion at the beginning of stance phase; K2: the biggest extension of the knee during a single stance; KTO: detaching the toes at the end of stance phase; K3: maximal knee flexion during swing phase; K4: maximal extension of the knee joint in the preparatory phase before stance phase. Values in bold are statistically significant.

Presented values obtained in the SB and DB ACLR groups refer to involved leg while values obtained in the control group refer to the right leg.

**Table 5:** Intragroup evaluation and intergroup comparison of examined variables in the hip joint.

Hip joint						
Internal (+) and external (−) rotation during gait cycle (degrees)						
Gait phases	Studied group	Involved/right leg		Uninvolved/left leg		*P* ^∗^
		*x *	SD	*x *	SD	
KHS	SB ACLR	2.19	7.30	−1.11	7.78	0.287
	DB ACLR	−3.88	11.20	−4.59	9.64	0.716
	Control	−7.27	6.26	−8.15	5.60	0.614
	*P *	**0.034**		0.095		

K1	SB ACLR	6.60	7.14	3.08	5.03	0.277
	DB ACLR	−0.55	10.46	0.15	8.59	0.770
	Control	−2.71	5.79	−3.95	5.72	0.440
	*P *	**0.023**		**0.042**		

K2	SB ACLR	7.18	6.25	6.16	8.11	0.768
	DB ACLR	1.68	9.41	2.98	7.10	0.547
	Control	−1.43	5.17	−0.11	4.65	0.415
	*P *	**0.020**		0.075		

KTO	SB ACLR	8.19	8.14	5.90	7.80	0.500
	DB ACLR	2.25	10.48	2.78	6.18	0.847
	Control	−4.15	6.37	−4.65	4.91	0.765
	*P *	**0.004**		**<0.001**		

K3	SB ACLR	9.49	8.10	5.70	8.42	0.169
	DB ACLR	1.85	11.14	1.48	11.12	0.904
	Control	−4.65	6.18	−4.25	6.54	0.836
	*P *	**0.001**		**0.027**		

K4	SB ACLR	0.45	5.66	−3.78	8.22	0.285
	DB ACLR	−6.15	12.75	−6.82	9.92	0.811
	Control	−12.97	6.35	−12.65	5.10	0.856
	*P *	**0.003**		**0.023**		

ACLR: anterior cruciate ligament reconstruction; SB ACLR: group after single-bundle ACLR; DB ACLR: group after double-bundle ACLR; control: control group; *x*: arithmetic mean; SD: standard deviation; KHS: the first contact between the foot and the ground; K1: maximal knee flexion at the beginning of stance phase; K2: the biggest extension of the knee during a single stance; KTO: detaching the toes at the end of stance phase; K3: maximal knee flexion during swing phase; K4: maximal extension of the knee joint in the preparatory phase before stance phase; *P*: significance level (one-way Anova); *P* ^∗^: significance level (parametric *t*-test for dependent samples). Values in bold are statistically significant.

Presented values obtained in the SB and DB ACLR groups refer to involved leg while values obtained in the control group refer to the right leg.

**Table 6:** Intergroup comparison of examined variables in the hip joint (results of the post hoc test).

Tukey's test results (*P*)								
Gait phase	Studied group	Involved/right leg			Studied group	Uninvolved/left leg		
		SB ACLR	DB ACLR	Control		SB ACLR	DB ACLR	Control
KHS	SB ACLR	—	0.220	**0.026**	SB ACLR	—	0.539	0.080
	DB ACLR	0.220	—	0.552	DB ACLR	0.539	—	0.453
	Control	**0.026**	0.552	—	Control	0.080	0.453	—

K1	SB ACLR	—	0.099	**0.019**	SB ACLR	—	0.557	**0.038**
	DB ACLR	0.099	—	0.759	DB ACLR	0.557	—	0.253
	Control	**0.019**	0.759	—	Control	**0.038**	0.253	—

K2	SB ACLR	—	0.175	**0.015**	SB ACLR	—	0.488	0.062
	DB ACLR	0.175	—	0.491	DB ACLR	0.488	—	0.434
	Control	**0.015**	0.491	—	Control	0.062	0.434	—

KTO	SB ACLR	—	0.229	**0.003**	SB ACLR	—	0.464	0.001
	DB ACLR	0.229	—	0.125	DB ACLR	0.464	—	**0.009**
	Control	**0.003**	0.125	—	Control	**0.001**	**0.009**	—

K3	SB ACLR	—	0.104	**0.001**	SB ACLR	—	0.497	**0.024**
	DB ACLR	0.104	—	0.132	DB ACLR	0.497	—	0.214
	Control	**0.001**	0.132	—	Control	**0.024**	0.214	—

K4	SB ACLR	—	0.200	**0.002**	SB ACLR	—	0.631	**0.024**
	DB ACLR	0.200	—	0.125	DB ACLR	0.631	—	0.137
	Control	**0.002**	0.125	—	Control	**0.024**	0.137	—

ACLR: anterior cruciate ligament reconstruction; SB ACLR: group after single-bundle ACLR; DB ACLR: group after double-bundle ACLR; control: control group; *x*: arithmetic mean; SD: standard deviation; KHS: the first contact between the foot and the ground; K1: maximal knee flexion at the beginning of stance phase; K2: the biggest extension of the knee during a single stance; KTO: detaching the toes at the end of stance phase; K3: maximal knee flexion during swing phase; K4: maximal extension of the knee joint in the preparatory phase before stance phase. Values in bold are statistically significant.

Presented values obtained in the SB and DB ACLR groups refer to involved leg while values obtained in the control group refer to the right leg.

**Table 7:** Intragroup evaluation and intergroup comparison of examined variables in the pelvis.

Pelvis						
Internal (+) and external (−) rotation during gait cycle (degrees)						
Gait phases	Studied group	Involved/right leg		Uninvolved/left leg		*P* ^∗^
		*x*	SD	*x*	SD	
KHS	SB ACLR	3.38	2.44	4.69	2.95	0.333
	DB ACLR	5.42	5.48	4.40	3.55	0.628
	Control	5.11	3.82	6.11	3.57	0.544
	*P*	0.479		0.381		

K1	SB ACLR	2.57	1.81	3.55	2.31	0.366
	DB ACLR	4.42	4.38	3.35	4.15	0.551
	Control	3.52	3.07	4.93	3.20	0.310
	*P*	0.430		0.413		

K2	SB ACLR	−0.52	2.44	−1.39	3.09	0.553
	DB ACLR	−0.58	2.93	−3.37	5.43	0.167
	Control	−2.27	3.66	−1.48	2.76	0.549
	*P*	0.268		0.372		

KTO	SB ACLR	−3.19	2.54	−3.46	3.16	0.594
	DB ACLR	−3.25	3.86	−5.40	4.85	0.321
	Control	−5.57	3.27	−5.20	2.76	0.764
	*P*	0.119		0.410		

K3	SB ACLR	−2.96	2.65	−3.97	2.85	0.546
	DB ACLR	−2.72	4.31	−5.07	3.26	0.247
	Control	−4.79	2.89	−4.59	2.35	0.864
	*P*	0.225		0.654		

K4	SB ACLR	3.98	3.65	3.04	3.18	0.564
	DB ACLR	5.99	5.65	3.18	3.68	0.213
	Control	5.22	3.48	5.81	4.34	0.254
	*P*	0.555		0.240		

ACLR: anterior cruciate ligament reconstruction; SB ACLR: group after single-bundle ACLR; DB ACLR: group after double-bundle ACLR; control: control group; *x*: arithmetic mean; SD: standard deviation; KHS: the first contact between the foot and the ground; K1: maximal knee flexion at the beginning of stance phase; K2: the biggest extension of the knee during a single stance; KTO: detaching the toes at the end of stance phase; K3: maximal knee flexion during swing phase; K4: maximal extension of the knee joint in the preparatory phase before stance phase; *P*: significance level (one-way Anova); *P* ^∗^: significance level (parametric *t*-test for dependent samples).

Presented values obtained in the SB and DB ACLR groups refer to involved leg while values obtained in the control group refer to the right leg.
